# Phase I Clinical Research of Jejunal Interposition in Adenocarcinoma of the Esophagogastric Junction II/III Proximal Gastrectomy

**DOI:** 10.1155/2016/1639654

**Published:** 2016-10-19

**Authors:** Kai Tao, Jian-Hong Dong

**Affiliations:** Department of Minimal Invasive Digestive Surgery, Tumor Hospital, Shanxi Medical University, Taiyuan 030013, China

## Abstract

*Objective. *To investigate the feasibility and specific methods of single-tract jejunal interposition between esophagus and remnant stomach (ers-STJI) in adenocarcinoma of the esophagogastric junction (AEG) II/III proximal gastrectomy.* Methods.* 15 AEG II/III gastric cancer (GC) patients in phase T1-3N0M0 with tumor size <5 cm were selected and they underwent proximal gastrectomy with ers-STJI from August 2013 to August 2014.* Results. *All of the 15 patients successfully completed GC R0 proximal gastrectomy with ers-STJI and no operative death or no significant complication occurred; one patient had anastomotic inflammatory granuloma. The digestive tract reconstruction time was 29.5 ± 5.7 min; the intraoperative blood loss was 96.7 ± 20.2 mL, and the number of lymph node dissections was 21.3 ± 3.0; the postoperative flatus time was 48.2 ± 11.9 h; the average length of hospital stay was 10.7 ± 2.3 d, and the average hospital stay cost was 60 ± 3 thousands. All of the patients were followed up for 12 months, and their postoperative single food intake, body weight, hemoglobin, and albumin were all recovered to the preoperative levels.* Conclusions.* The applications of ers-STJI in proximal gastrectomy were safe and feasible, and the length of jejunal interposition could be 15–25 cm.

## 1. Introduction

Since Siewert and Stein [1], German scholars, classified adenocarcinoma of the esophagogastric junction (AEG) into AEG I/II/III for the first time in 1998, AEG has obtained extensive attention and in-depth researches. In 2009, the National Comprehensive Cancer Network (NCCN) (USA) [2] treated AEG as a separate disease and gave it clear definition and division. Although the JCOG9502 study in Japan confirmed that the transabdominal approach was better than the trans-left thoracoabdominal approach toward AEG II/III [3], the range of surgical resection and the method of digestive tract reconstruction (DTR) are still the major disputes of AEG currently. Early surgeries used proximal gastrectomy with esophagus-residual stomach anastomosis, due to its higher incidence of regurgitation; the Japanese Gastric Cancer Association recommended the standard operation to be total gastrectomy with esophagus-jejunal Roux-en-Y anastomosis D2 surgery in 2011 [4], but it still had such problems as damaging the continuity of digestive tract, affecting nutrient absorption, and dumping syndrome, which was prone to causing postoperative weight loss, anemia, and other complications [5, 6]. However, proximal gastrectomy could retain gastric remnant and duodenum, so patients' postoperative body weight, hemoglobin, albumin, cholesterol, and other nutritional statuses would be better than total gastrectomy [7]. Therefore, proximal gastrectomy has obtained attention and discussion again [8]. Domestic and foreign scholars had carried out the investigations of ers-STJI [9], but the specific operation methods and the reconstruction data were still controversial. In this study, we explored the feasibility of jejunal interposition after proximal gastrectomy in patients with early AEG II/III cancer and its specific reconstruction methods.

## 2. Materials and Methods

### 2.1. Information

15 AEG II/III patients were selected from August 2013 to August 2014, including 14 males (49–70 years old) and 1 female, with the mean age of 60.5 ± 6.1 years old (Table 1). Inclusion criteria include the following: (1) the patient should be positively preoperatively diagnosed as AEGII/III (in Siewert II cases we had chosen, the upper bound was below the dentate line and the center of tumors was located within the dentate line and below the range of 2 cm); (2) the residual stomach should be equal to or more than 1/2 after the tumor was resected according to the requirements; (3) tumor range must be <5 cm, and preoperative computed tomography (CT) and echoendoscopy confirmed the tumor was not in local advanced stage; (4) chest X-ray, abdominal ultrasound, and abdominal CT showed no distant metastasis; (5) electrocardiogram (ECG), lung functions, blood routine, and hepatonephric functions were normal. This study was approved by the hospital ethics committee, and all the patients signed the informed consent (Table 1). This study was conducted in accordance with the Declaration of Helsinki. This study was conducted with approval from the Ethics Committee of Shanxi Medical University. Written informed consent was obtained from all participants.

### 2.2. Surgical Operation

**Figure 1:**
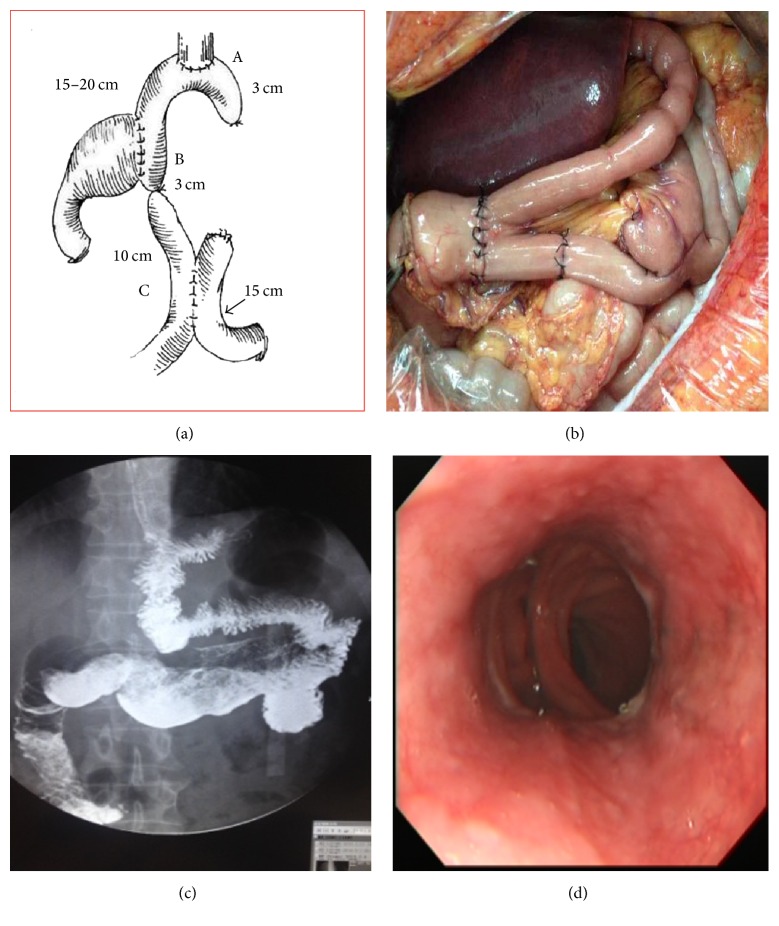
Postoperative results. (a) Schematic diagram. (b) Restitution. (c) Visualization. (d) Gastroscope.

First, there is routine incision and entering the abdomen, and then the greater and the lesser omentum were freed and incised; numbers 4 d, sb, sa, 10, and 2 lymph nodes were dissected in turn along the greater curvature, and the left gastric omentum and the short gastric vessel were then amputated; numbers 12a, 8a, 7, 9, and 1 lymph nodes were then dissected, and numbers 5 and 6 lymph nodes were swept separately and simultaneously. After suturing and cutting off the left gastric artery, number 3 lymph nodes were dissected to isolate the belly esophagus and to cut off the left and right vagus nerve. The esophagus was then cut off 3~4 cm away from the proximal end of the tumor; one 4 cm stomach wall was then clamped vertically using one Kocher clamp at the proposed cut-off site along the greater gastric curvature; the specimen was then closed and cut off along the lesser gastric curvature side using a straight cutting-stitching instrument, and about 50% to 60% of the proximal stomach tissues were then resected, with the stomach incision edge 3~5 cm away from the tumor. The residue greater gastric curvature was then prepared into 4 cm tube for the anastomosis. The jejunum and mesentery were then cut off 25–35 cm away from the distal end of Treitz ligament. The distal ends of 1 to 2 secondary jejunal branch arteries were then cut off according to the specific situations, but the enteric vessel bow and marginal vessels were retained to prepare the bridge loop, which was put up from the front or rear site of transverse colon. One number 26 round stapler was then used to perform esophagus-jejunum anastomosis and to close the jejunum stump (the indwelled caecum should not exceed 3 cm) 3~5 cm away from the distal end of jejunum; 15~25 cm away from this anastomotic stoma, one number 26 round stapler was used to perform the side to side anastomosis of gastric remnant (rear wall) and jejunum, and the jejunum 3 cm from the distal end of this anastomotic stoma was moderately ligatured and blocked using one thick silk. The proximal and distal jejunum side-side anastomoses were then performed at the site 10 cm away from the stomach-jejunum anastomosis to close the proximal jejunal stump and mesangial gap. After placing the gastrointestinal decompression tube and nutrient tube via the stomach stump, the stump was closed with one peritoneal drainage tube indwelled. The results were shown in Figure 1.

### 2.3. Observation Indexes

DTR time (min), postoperative ventilation time (h), length of hospital stay (d), and hospitalization cost (ten thousands) and such complications as anastomotic leakage, bleeding, infection, obstruction, reflux esophagitis, anastomotic stenosis, and dumping syndrome were also observed.

The (1) classification of gastroesophageal reflux symptoms (Visick classification), (2) classification of endoscopic reflux symptoms (Los Angeles, LA), and (3) gastrointestinal symptom rating scale (GSRS) were used to statistically analyze the incidences of reflux, vomiting, heartburn, and swallowing difficulty to evaluate the postoperative efficacies. The changes of single food intake and body weight before the surgery and 12 months after the surgery were recorded, and the changes of serum gastrin, albumin, and hemoglobin were recorded to evaluate the one-year postoperative quality of life.

*Note*. Regarding Los Angeles (LA), grade A, the esophageal mucosa had one or several mucosal injuries with the long diameter <5 mm; for grade B, besides the same symptoms as grade A, the long diameter of the continuous lesions' mucosal injury was >5 mm, but the injuries on each mucosal fold were discontinuous; concerning grade C, at least one mucosal injury exhibited the continuity of 2 folds or more but less than 3/4 of the perimeter; for grade D, the mucosa had circumferential fusion injury, which was more than 3/4 of the perimeter. Regarding Visick, we have the following classification: grade I, asymptomatic; grade II, with occasional symptoms; grade III, with obvious but tolerable symptoms; and grade IV, with obvious and intolerable symptoms. About GSRS, the assessment items included choking, bloating, dumping syndrome, vomiting, or decreased appetite. The classification is as follows: 0 points, asymptomatic; 1 point, mild symptoms; 2 points, moderate symptoms; and 3 points, severe symptoms.

## 3. Results

### 3.1. Surgical Data

The jejunum and mesentery of the patients in this study were cut off at 25–35 cm away from the distal end of Treitz ligament, with the average distance from the second jejunal branch artery to the Treitz ligament as 31.5 ± 3.2 cm and jejunum-esophageal anastomotic stump as <3 cm; the jejunum-remnant stomach anastomosis was performed 15–25 cm away from this stoma, and the jejunum was closed and established one single tract 3 cm away from the gastrointestinal anastomotic stoma; the average DTR time was 29.5 ± 5.7 min, and the postreconstruction angle between the jejunal afferent loop and gastric remnant was 120°–180°. The intraoperative blood loss was 96.7 ± 20.2 mL, and 21.3 ± 3.0 lymph nodes were dissected; numbers 5 and 6 lymph nodes had no metastasis.

### 3.2. Postoperative Recovery and Complications

The average postoperative ventilation time was 48.2 ± 11.9 h, the average hospital stay was 10.7 ± 2.3 d, and the average hospitalization cost was 60 ± 3 thousands. No anastomotic leakage, bleeding, infection, obstruction, or dumping syndrome occurred, and one case had anastomotic inflammatory granuloma and was cured after treatment. The pathological staging revealed eight cases of T1N0M0, three cases of T2N0M0, three cases of T3N0M0, and one case of T2N1M0; no chemotherapy was performed.

### 3.3. Evaluation of Postoperative Efficacies

For Visick grading, there were 14 cases of grade I and 1 case of grade III; for LA, there were 12 cases of grade A, 2 cases of grade B, and 1 case of grade C; for CGSRS, there were 10 cases of 0 points, 4 cases of 1 point, and 1 case of 2 points, with the mean as 0.33 points.

### 3.4. One-Year Follow-Up

**Figure 2:**
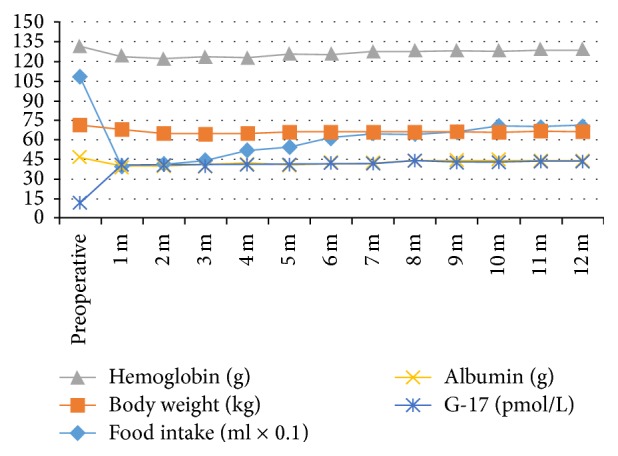
Change trends of indexes 1–12 months after the surgery.

The average postoperative 1-month single intake was 393.3 mL, which was increased to about 700 mL and stabilized from the 4th~6th month but averagely reduced by 373.3 mL in the 12th month compared to that before the surgery. The postoperative 1-month body weight was averagely reduced by 3.33 kg than preoperatively, which was gradually reduced to the lowest point within the next three months and reduced by 6.66 kg than preoperatively; from the 4th month, the body weight steadily rose up and stabilized 6 months later but averagely reduced by 4.4 kg in the 12th month than preoperatively. Gastrin G-17 was increased postoperatively to a higher level and remained stable. Hemoglobin was reduced postoperatively and gradually recovered near to the preoperative level in 2-3 months. Albumin exhibited temple postoperative reduction and gradually recovered near to the preoperative level in 1 month (Figure 2). There was no recurrence and death case.

## 4. Discussion

GC surgeries need to consider such issues as radical treatment, reconstruction, and functions. This study showed that all of the patients had no number 5 or number 6 lymph node metastasis. Brar et al. [10] studied the feasibility of modified D2 surgery toward tumors along the lesser gastric curvature, and the results showed that the postoperative lymph node dissection and prognosis in the D1+ patients without lymph node dissection around the splenic hilum, pancreatic surrounding, and hepatoduodenal ligament had no significant rate difference, but the postoperative complications were significantly reduced. The 13th edition of Guidelines for the Treatment of Gastric Cancer in Japan has classified numbers 5, 6, 12a, 12b, and 14v lymph nodes into the D3 lymph nodes of AEG [11]. Therefore, it was feasible to perform limited proximal gastrectomy D1+ surgery toward AEGII/III GC patients [12, 13].

The R0 resection-based reconstruction methods are the main reasons that would impact the postoperative functions. Proximal gastrectomy retains partial distal stomach and its functions, and the main reconstruction methods currently used are esophagogastrostomy (anterior and posterior stomach walls) [14] and jejunal interposition (single tract, dual tract, and interposition with jejunal pouch). Japan performed statistics toward 145 hospitals in 2012 [9], and the results showed that, after proximal gastrectomy, 48% of the patients underwent esophagogastrostomy, 28% underwent jejunal interposition, 13% underwent dual-tract anastomosis, and 7% underwent jejunal interposition pouch. 46% of the patients had the length of jejunal interposition as 10 cm, and 28% had it as 15 cm. The length of jejunal interposition is the most important issue that needs to be considered during this surgical method. Too short indwelling not only would cause poor antireflux effects but also would increase the tension at gastrointestinal anastomotic stoma, thus increasing the incidence of such complications as anastomotic leakage and so forth. However, too long jejunal interposition would increase food's entering-remnant-stomach distance, thus causing the possibility of food retention. The length of jejunal interposition in this study was 15–25 cm, and the postreconstruction angle between the jejunal afferent loop and gastric remnant was 120°–180°, so food could fluently enter the remnant stomach through the jejunal afferent loop; if the length of jejunal interposition is too long, it would be bound to increase or decrease this angle, thus increasing the resistance for food to enter the gastric remnant, followed by choking or small amount of single food intake. Therefore, according to the size of remnant stomach, the length of jejunal interposition should be kept within 15–25 cm so as to ensure appropriate tension and angle for the feasibility of this surgery.

The lengths of proximal jejunum and distal jejunum kept for “Y”-shape anastomosis should be decided according to the distance between the second jejunal artery branch and Treiz ligament. In this study, the average distance in the 15 patients was 31.5 ± 3.2 cm; too short length would affect the blood supply toward proximal jejunum, so there was no need for single-tract reconstruction to extremely emphasize the indwelling distance of proximal jejunum because a little longer length would not cause any effect. The side-side anastomotic site of jejunum-jejunum was normally located 10 cm away from the gastrointestinal anastomotic stoma, at which the general intestine-intestine anastomotic angle was mostly 60°–90°, exhibiting an antegrade state, so the distance through which food should pass would be the shortest and most smooth. The site selected for single tract should ligate the jejunum 3 cm away from the stomach-jejunum anastomotic stoma, and this distance would not be too short so as to cause anastomotic stenosis or too long so as to cause partial food retention.

After proximal gastrectomy, distal gastric functions would be another issue that should be considered, and there are two opinions toward this issue, namely, vagus nerve reservation and pyloroplasty. The main purpose of pyloroplasty is to prevent food retention inside the remnant stomach, but it would be prone to causing bile reflux. One cohort study that reserved the vagus nerve but did not perform pyloroplasty [15] and one long-term randomized controlled study of gastric resection that did not reserve the vagus nerve but performed pyloroplasty [16] prompted good pyloric functions. However, the operations of reserving the vagus nerve are complex, and there exists the risk of incomplete lymph node dissection. All of the patients in this study reserved about 50% of distal stomach and did not reserve the vagus nerve and did not undergo pyloroplasty, and no retention case occurred, indicating that when 1/2 or more gastric remnants could be reserved, the gastric emptying would not be affected basically, so there was no need of pyloroplasty or retaining the vagus nerve.

The postoperative endoscopy and GSRS in all of the patients showed no occurrence of complications such as significant reflux, and all indicators were within reasonable limits; all of the patients were discharged fluently, and no death occurred. The postoperative single food intake, body weight, hemoglobin, and albumin exhibited the same changing trend, which were transiently decreased and then slowly recovered to near normal level, indicating the importance of single food intake and feasibility of this reconstruction method. Meanwhile, we found that the gastrin G-17 level was significantly increased compared to that before the surgery instead of being decreased, and its mechanisms were still not clear. G-17 has important roles in gastrointestinal hormones [17], and this also demonstrated the necessity of retaining the gastric antrum.

Besides the roles of pouch, the approach of single-tract jejunal interposition that retains the distal stomach also has the following advantages: (1) the presence of pylorus could fully mill and stir food, thus profiting the absorption of nutrients and avoiding the occurrence of dumping syndrome [18]; (2) it retains the storage roles of stomach so as to ensure a bigger single food intake; (3) the intestinal peristalsis could be ensured smoothly; furthermore, normal tracts are retained, so when food passes through the duodenum, it could stimulate the duodenum to secrete cholecystokinin and pancreozymin, thus promoting the bile to be discharged into the gut and the pancreatic juice to be secreted, as well as helping the digestion and absorption of food, fat, calcium, iron, vitamin B12, or carbohydrates [19]; (4) it could retain most of secretory functions of stomach, so the impacts on gastrointestinal endocrine functions could be minimized, and the incidence of gastrointestinal dysfunction could be reduced [20]; (5) most importantly, it could avoid the occurrence of reflux esophagitis, during which jejunal interposition could play a buffer role.

However, this study did not elucidate the motivation of distal stomach and impacts on the secretion of pepsin after the proximal stomach resection, and it still remained to be further studied.

In summary, proximal gastrectomy with reasonable jejunal interposition was safe and convenient for the treatment of SAEII/III, so it might possibly provide a better solution than DTR for the treatment of AEG. Next, we would conduct the phase II clinical study and compare the efficacies with esophagogastrostomy and total gastrectomy after proximal gastrectomy so as to further clarify the occurrence of various surgical complications, as well as the impacts on long-term life qualities and gastrointestinal secretions.

## Figures and Tables

**Table 1:** Clinical data of the patients.

Patient	Gender	Age	AEG typing	Intraoperative blood loss (mL)	Positive rate of number 5 and number 6 lymph nodes	DTR (min)	Postoperative ventilation time (h)	Average length of hospital stay (days)
1	M	49	II	100	0	36	39	10
2	M	64	II	120	0	25	53	11
3	M	59	II	60	0	31	66	12
4	M	68	II	80	0	26	56	10
5	M	53	II	120	0	28	42	9
6	M	42	III	100	0	30	37	11
7	M	70	II	150	0	35	55	10
8	M	62	II	80	0	22	71	11
9	M	60	II	80	0	38	56	9
10	M	65	II	100	0	27	60	12
11	M	66	II	60	0	25	38	15
12	M	61	II	80	0	25	40	9
13	M	62	II	100	0	33	36	10
14	M	58	II	100	0	32	33	12
15	F	69	II	120	0	30	41	10

$\overset{-}{x}\pm s$		60.5 ± 6.1		96.7 ± 20.2		29.5 ± 5.7	48.2 ± 11.9	10.7 ± 2.3
